# Biochar/Kevlar Nanofiber Mixed Matrix Nanofiltration Membranes with Enhanced Dye/Salt Separation Performance

**DOI:** 10.3390/membranes11060443

**Published:** 2021-06-12

**Authors:** Shiguo Gu, Lei Li, Fei Liu, Jian Li

**Affiliations:** 1 Laboratory of Environmental Biotechnology, School of Environmental and Civil Engineering, Jiangnan University, Wuxi 214122, China; ychgushiguo@163.com (S.G.); ll19980913@163.com (L.L.); 2 State Key Laboratory of Food Science and Technology, Jiangnan University, Wuxi 214122, China; 3 School of Food Science and Technology, Jiangnan University, Wuxi 214122, China

**Keywords:** biochar, Kevlar nanofiber, mixed matrix membrane, nanofiltration, dye/salt separation

## Abstract

Mixed matrix membranes have received ever-growing attention due to their high separation performance, taking the advantages of both porous fillers and polymer backbones. However, limitations still exist due to the instability of polymers in harsh environments. Here, Kevlar aramid nanofibers, a nanoscale version of poly(paraphenylene terephthalamide), were applied to fabricate a nanofiltration membrane by a thermo-assisted phase inversion method due to their high mechanical strength, physical stability and resistance to solvents. Biochar was incorporated in the Kevlar nanofibers to evaluate its performance in dye/salt separation performance. The fillers’ distribution in the polymeric matrix, structural characteristics, and the interaction of fillers with the polymer in the membrane were characterized via SEM, FTIR, AFM and contact angle analysis. Under the optimal fabrication conditions, the obtained membrane exhibited a pure water flux of 3.83 L m^−2^ h^−1^ bar^−1^ with a dye rejection of 90.55%, 93.54% and 95.41% for Congo red, methyl blue and Reactive blue 19, respectively. Meanwhile, the mixed matrix membrane maintained a salt rejection of 59.92% and 85.37% for NaCl and Na_2_SO_4_, respectively. The obtained membrane with high separation performance suggested that Kevlar nanofiber and biochar are good candidates for membrane synthesis.

## 1. Introduction

Membrane separation technology has been extensively used in recent years for the treatment of wastewater due to its high separation efficiency and easy operation [1,2]. The most typical polymers used in preparing existing membranes include polyvinylidene fluoride (PVDF), polypropylene (PP), and polytetrafluoroethylene (PTFE) et al., which are common engineering plastics that can be readily processed into microfiltration (MF) and ultrafiltration (UF) membranes [3,4]. Microporous membranes prepared with organic polymers exhibit excellent permeability and thermal stability, which can maintain good selectivity towards high molecular-weight molecules due to the networks formed by the rigid polymer chains [5,6,7]. UF membranes, with a pore size around 10–100 nm, allow macromolecules (i.e., dye, suspended particles, and natural organic matter) and dissolved solids (i.e., inorganic salts) to pass through. However, those membranes suffer from the drawbacks of poor structural geometry of pores, macro-void formation and wide pore size distribution. Besides, due to the limited rejection of UF/MF membranes towards salts, their application is restricted in many industrial processes, such as the metallurgical industry, textile industry and heavy metal resource recovery [8].

Nanofiltration (NF), with the properties between UF and reverse osmosis (RO), possesses a pore size between 1 and 10 nm, which can separate target molecules with a molecular weight between 300 Da and 500 Da. The separation process of NF membranes is decided by the size-sieving and electrostatic effect. A typical NF membrane is constructed by an interfacial polymerization method based on an MF/UF substrate. However, previous study has revealed that the differences in physical and chemical properties for different substrates could lead to nanofiltration with distinct separation performance [9]. Currently, the polymeric substrates used for NF membranes include polysulfone (PSf) [10], polyacrylonitrile (PAN) [11], polycarbonate (PC) [12], and polyethersulfone (PES) [13]. However, for some specific applications, such as acid recovery and solvent nanofiltration, the stability of the above mentioned substrates is still a challenge [14]. In addition, the formation of a polyamide skin layer by an interfacial polymerization step makes the membrane fabrication progress much more complex. Thus, novel materials with enhanced stability and a simple synthesis procedure are appealing for NF membrane fabrication.

Kevlar aramid nanofibers (KANFs) are kinds of one-dimensional polymer nanomaterials, which are composed of the nanoscale version of poly (paraphenylene terephthalamide) (PPTA) molecules [15]. KANFs have superior mechanical strength, good thermal stability as well as excellent temperature and solvent resistance [16] due to the strong intermolecular bonding (e.g., π–π stacking and hydrogen bonding) interactions in PPTA polymer chains [17,18,19]. Therefore, the application of KANFs as a membrane substrate is expected to impose special characteristics and high separation performance on NF membranes. Currently, researchers have successfully applied inorganic material-doped aramid nanofibers as in situ nanoscale building blocks to form high-performance composite NF membranes. For example, Yang et al. manufactured a composite KANF-based membrane by incorporating FeOOH and ZnO nanoparticles (NPs) [20]. The obtained membranes possessed excellent tensile and wear resistance properties, which can be used to separate oil-in-water and water-in-oil emulsions. Liu et al. developed composite KANF membranes with cadmium telluride (CdTe) nanocrystals and phosphoric acid (PA) as additives by a spin coating method [21]. The accumulation of Kevlar nanofibers could support the layered membrane while CdTe nanocrystals served as the bridges to link PA molecules with Kevlar nanofibers, besides improving the mechanical properties. The satisfactory mechanical properties guaranteed the prepared membranes’ stability while working at high temperatures. Despite the fact that numerous studies have been conducted to prepare Mixed Matrix Membranes (MMMs) with Kevlar and inorganic nano-filters, little is known about Kevlar combining with organic materials to form composite NF membranes. Thus, further studies should be continued to reveal the possibility of fabricating high-performance MMMs by Kevlar and organic materials.

Currently, studies on the applications of carbon-based nano-materials (e.g., graphene and derivative graphene oxide (GO)) in fabricating composite NF membranes have been widely reported. The novel GO-based NF membrane showed high efficiency in heavy metal removal [12], desalination [22,23], and organic dye removal [24]. The membrane assembled by both GO and multiwalled carbon nanotubes (MW-CNTs) exhibited an excellent permeance with a water permeability of 11.3 L m^−2^ h^−1^ bar^−1^, which was more than twice that of the neat GO NF membrane. Meanwhile, a GO/MW-CNT-modified membrane maintained a high dye rejection (>99% for Direct Yellow and >96% methyl orange) [25]. Compared to GO and CNTs, nano biochar (nano-BC) has the potential to serve as a promising candidate for membrane synthesis due to its easy accessibility and high mechanical and chemical stabilities [26,27,28]. Simultaneously, nano-BC possesses high surface reactivity, porosity, a dense nanopore structure, and a superhydrophobic structure due to its highly negatively charged surface and large oxygen containing groups. Hence, BC with promising chemical and physical properties could provide extra channels for the water permeance of the MMMs. Consequently, the combination of nano-BC and Kevlar could be an alternative to synthesize novel NF membranes with enhanced stability and alleviate the trade-off relationship between the separation efficiency and water flux.

In this work, the separation performance of the composite NF membrane synthesized by incorporating nano-BC into a Kevlar nanofiber substrate supported on a non-woven fabric has been studied. The characterization of the composite NF membrane was conducted by a suite of spectroscopic technology including X-ray photoelectron spectroscopy (XPS), Fourier transform infrared spectroscopy (FTIR), contact angles (CA), dynamic light scattering (DLS), scanning electron microscopy (SEM), and atomic force microscopy (AFM). To evaluate the flux and separation efficiency of the composite NF membrane, with pure water flux and salt, dye rejection tests were investigated. This research is expected to provide some guidance for the synthesis of MMMs by Kevlar nanofibers or BC materials and lay a foundation for further applications of Kevlar or BC-based membranes.

## 2. Materials and Methods

### 2.1. Materials

Bulk Kevlar 69 was purchased from The Thread Exchange, Inc., USA (DuPont, Wilmington, DE, USA). Sodium chloride (NaCl, 99%), sodium sulfate (Na_2_SO_4_, 99%), and potassium hydroxide (KOH, 90%) were purchased from Sinopharm Chemical Reagent Co., Ltd. (Shanghai, China). Dimethyl sulfoxide (DMSO, ≥99%) was obtained from Shanghai Titan Scientific Co., Ltd (Shanghai, China). Congo red (Mw 696.66 Da, negative charge), methyl blue (Mw 799.80 Da, negative charge), and Reactive blue 19 (Mw 626.54 Da, negative charge) were purchased from Shanghai Titan Scientific Co., Ltd (Shanghai, China). Non-woven fabric was provided by Tianlue Textile New Material Co., Ltd (Shanghai, China). Deionized (DI) water was used throughout the experiments.

### 2.2. Synthesis of Nano Biochar (Nano-BC)

Wood was selected as the raw material for biochar production. Before use, the raw material was rinsed several times with DI water to remove surface impurities. After being oven-dried at 80 °C, the material was crushed and passed through a 2.0 mm mesh. Then, the powdered material was fed into a tubular reactor within a muffle furnace and slowly pyrolyzed (10 °C/min) in a N_2_ atmosphere at 550 °C for 120 min. The extraction method of nano-BC was the same as our previous work [27,28].

### 2.3. Preparation of Composite NF Membranes

The casting solution (Kevlar nanofiber) was obtained by dissolving 4 g bulk Kevlar 69 and 2.0 g KOH in 200 mL DMSO/DI water (volume ratio 40:1) and stirred at room temperature for two weeks until a dark red viscous homogeneous solution was formed. To form nano-BC/Kevlar nanofiber solutions, nano-BC (2.5%, 5%, 7.5%, 10%, mass ratio) was added to the casting solution, which was stirred for 24 h and allowed to stand for 3 h for defoaming. The nano-BC/Kevlar nanofiber solutions were cast onto a glass plate by a casting knife with a certain gap (100, 150, and 200 μm). After that, the liquid membrane was placed in an oven for specific time at 60 °C and transferred to DI water for a water bath. After standing for 1 h, the nano-BC/Kevlar nanofiber membrane was lifted with non-woven fabric and transfer to pure DI water for further application.

### 2.4. Characterization of Composite NF Membranes

The morphologies of the composite NF membranes were inspected using scanning electron microscopy (SEM, Zeiss Gemini 300, Germany) and atomic force microscopy (AFM, Bruker Dimension Edge, USA). Surface chemical composition of membranes and nano-BC was observed using X-ray photoelectron spectroscopy (XPS) (Thermo Scientific K-Alpha, USA). The functional groups (composition of membranes and nano-BC) were recorded by Fourier transform infrared spectroscopy (FTIR) (IRTracer-100, Shimadzu, Japan). The samples were scanned from 400 to 4000 cm^−1^ by using a FTIR with a resolution of 5 cm^−1^. The contact angles (CA) of membrane samples were assessed using a KSV CA meter (CAM200) equipped with CA instrument and software (Kruss, Germany) at room temperature. The molecular weight cut-off (MWCO) of the MMMs was identified by the filtration of polyethylene glycol (PEG) with different molecular weights (200 ppm, 200 Da, 400 Da, 600 Da, 1000 Da, 1500 Da, and 2000 Da). The intensity-weighted hydrodynamic diameter (Dh) of Kevlar and nano-BC was measured using dynamic light scattering (DLS) of a Zetasizer (ZEN3600, Malvren, UK) at a scattering angle of 173°. Moreover, zero point of charge (ZPC) of all samples was obtained by determining zeta potentials of the samples as a function of solution pHs using DLS analyzer.

### 2.5. Permeability and Salt/Dye Rejections for Composite NF Membranes

The separation performance of the composite NF membranes, including the pure water permeability and rejections to dyes/salts, was estimated by a typical cross-flow circulation system with an active membrane area of 7.065 cm^2^. Initially, to stabilize the membrane before testing the performance, pre-pressure was carried out for 2 h under 6 bar. The feed solutions included DI water, saline solutions (1 g/L) containing NaCl and Na_2_SO_4_, and dye solution (0.2 g/L) including Congo red, methyl blue, and Reactive blue 19. The experiments were conducted under a pressure of 4 bar at room temperature. The salt concentrations were measured with an electrical conductivity meter (SevenExcellence, China) and the concentration of dyes was analyzed by UV–Vis spectroscopy (Shimadzu, Japan) at the maximal absorption wavelength of the organic dyes.

DI water flux (J, L m^−2^ h^−1^ bar^−1^) was calculated using the following Equation (1):

(1) $$J=\frac{V}{S\times H\times P}$$

where V (L), S (m^2^), H (h), and P (bar) are the volume of the permeated water (L), the effective area of the membrane (m^2^), the permeation time (h), and the operating pressure (bar), respectively.

The rejection ratios (R) of the membranes were defined by Equation (2):

(2) $$R=1-\frac{C_{1}}{C_{2}}\times 100\%$$

where C_1_ (mg/L) and C_2_ (mg/L) are the concentrations of dyes and salts in the permeance and the feed sides, respectively.

## 3. Results

### 3.1. Nano-BC Properties

**Figure 1:**
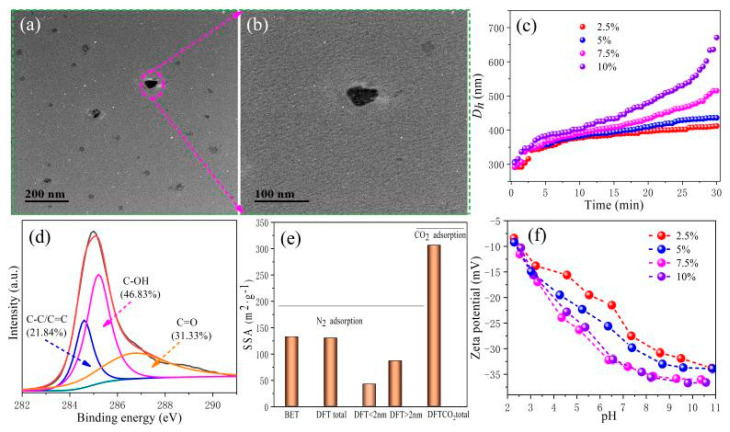
Characterization of nano-BC. (**a**,**b**) TEM images, (**c**) Aggregation profiles of nano-BC in DI water, (**d**) High-resolution XPS C 1s spectra, (**e**) Specific surface area (SSA) according to N_2_ and CO_2_ gas adsorption, and (**f**) ζ potential as a function of pH.

The physical and chemical properties of BC determine the performance of the obtained MMMs. Thus, the features of the BC should be extensively explored by a series of characterizations. As depicted in Figure 1a, TEM images revealed that the selected nano-BC is in the nanoscale with particle sizes around ∼60 nm, which is consistent with previous observations [27,29]. Meanwhile, the hydraulic diameter (D_h_) of nano-BC was also measured and the D_h_ of nano-BC was revealed around 300 nm (Figure 1b). The TEM and D_h_ characterizations of BC indicated that the D_h_ of particles significantly increased when BC was in a water solution. This can be explained by the hydration shell of water molecules forming around the nano-BCs [30]. In addition, as revealed in Figure 1b, the value of D_h_ increased with time, which indicates that a serious agglomeration phenomenon of nano-BC occurred when nano-BC was in the water solution. Thus, the dispersion of nano-BC is crucially important for the fabrication of NF MMMs.

The functional groups present on the surface of nano-BC were examined by XPS. The C 1s spectra can be deconvoluted into three major carbon functional groups at C–C/C=C (284.6 eV), C–OH (285.2 eV), and C=O (286.7 eV), respectively [31,32]. Lian et al. reported that the surface activity (polarity index and hydrophilicity) of nano-BC (rice straw) is higher than that of bulk biochar [27]. Here, nano-BC has a high content of O-containing groups including hydroxyl (46.83%) and carboxyl (31.33%), which indicates the high polarity and surface charge density. The elemental composition, atomic ratio, and polar index of the nano-BC are listed in Table 1. The high polarity index [(O + N)/C] and hydrophilicity index [O/C] of nano-BC confirms the presence of abundant polar functional and hydrophilicity groups. The specific surface area (SSA) of nano-BC obtained by CO_2_ adsorption is 306.4 m^2^/g, which is 131.89% higher than that by N_2_ adsorption (Figure 1d). The micropore of biochar accounts for 31.82% of the total pore by N_2_ adsorption. These results reveal that nano-BC has distinct physicochemical properties (micropore, hydrophilicity, and aggregation). The ζ potential of nano-BC as a function of pH is shown in Figure 1e. In a wide pH range from 2 to 11, the nano-BC is negatively charged, which is mainly due to the deprotonation of the carboxyl group at the edges of nano-BC nanosheets. These superior features provide nano-BC with superb traits for membrane fabrication.

### 3.2. Membrane Characterization

#### 3.2.1. FTIR of the Membrane

**Figure 2:**
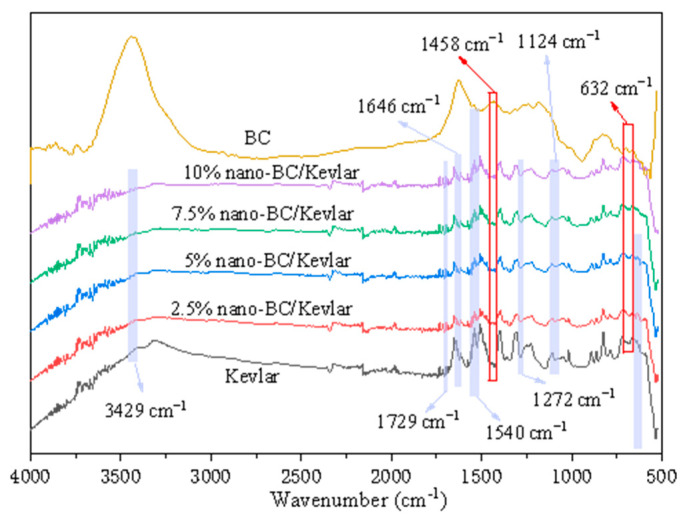
FTIR spectra of nano-BC, Kevlar fiber and the nano-BC/Kevlar MMMs.

The characteristic, unambiguous positions of the main functional groups of Kevlar were C=O, N–H, C–N, and phenyl (Figure 2) [33]. The existence of peaks at 3429 cm^−1^ and 1540 cm^−1^ was N–H stretching from the Kevlar [34]. The peaks appearing at 1729 cm^−1^ and 1646 cm^−1^ were caused by the group of C=O in the Kevlar structure [35]. Peaks at 1272 cm^−1^ and 1124 cm^−1^ were ascribed to the phenyl–N vibration and C–N stretching, respectively [36]. The FTIR spectrum of BC around 632 cm^−1^ and 1458 cm^−1^ could be the –OH vibration. C–H in-plane bending vibration occurs at the BC interface (Figure 2) [37]. The nano-BC/Kevlar MMMs showed the corresponding absorption peaks of –OH and this confirmed that nano-BC was successfully introduced into the structures of nano-BC/Kevlar.

#### 3.2.2. Surface Morphology of the Membrane

**Figure 3:**
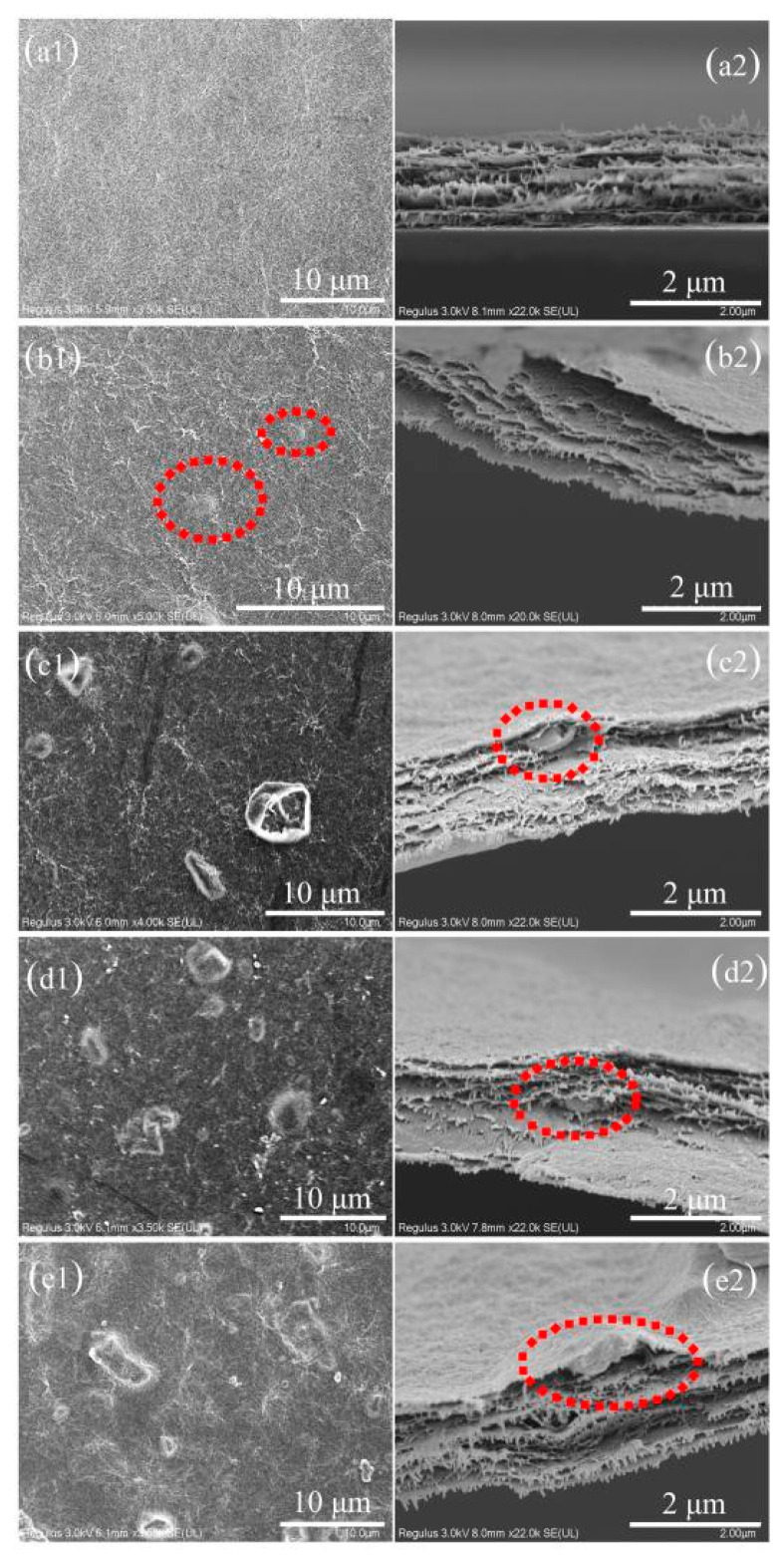
SEM observations of membrane surface (**a1**–**e1**) and cross-sectional morphology (**a2**–**e2**): (**a1**,**a2**) Kevlar, (**b1**,**b2**) 2.5% nano-BC/Kevlar, (**c1**,**c2**) 5% nano-BC/Kevlar, (**d1**,**d2**) 7.5% nano-BC/Kevlar, and (**e1**,**e2**) 10% nano-BC/Kevlar.

The surface and cross-sectional morphologies of different nano-BC/Kevlar membranes were observed by SEM, as shown in Figure 3. From Figure 3a1,a2, it is obvious that the Kevlar membrane surface exhibited a dense and smooth surface with fiber shape-like morphology atop after heating treatment and phase inversion [17]. Cross-sectional images confirmed the dense skin layer with a layered structure inside (Figure 3b1,b2), which is the typical structure of a Kevlar-based membrane [38]. Compared with pure (Kevlar) membranes, the morphology of composite (nano-BC/Kevlar) membranes is changed significantly by the addition of nano-BC. Overall, the MMMs sustained the fiber structure. However, numerous bulges with nano/micro-particle shapes were found on the membrane surface (Figure 3b1–e1) after decoration with nano-BC. With more nano-BC introduced to the Kevlar membrane, the membrane surface became rougher. More peaks and valleys were also observed. The reason for the appearance of a micro-mountain structure on the surface of the membrane is due to the inevitable aggregation of nano-BC [39,40]. From the cross-section images of the MMMs (Figure 3b2–e2), nano-BC was observed at different positions of the membrane and the BC was fully covered by the Kevlar fiber. With more BC loading, the membrane becomes much looser and more BC can be found. The obtained membrane with BC as additives can provide extra channels for molecular transfer while the Kevlar fiber maintains a high rejection, which provides great potential for enhancing the membrane separation performance.

#### 3.2.3. AFM Results of the Membrane

**Figure 4:**
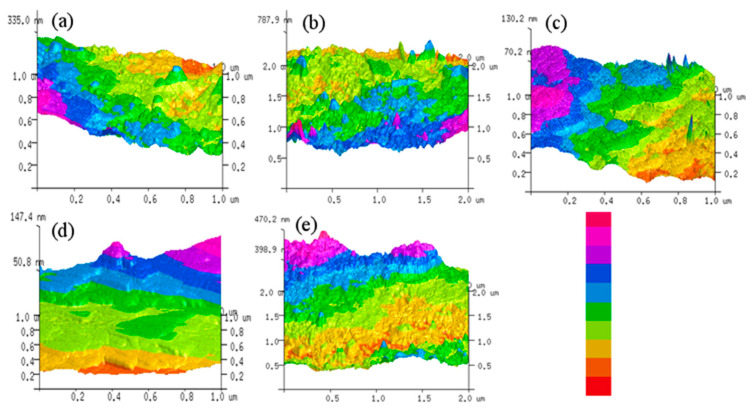
AFM images of the primary and the modified membranes. (**a**) Kevlar, (**b**) 2.5% nano-BC/Kevlar, (**c**) 5% nano-BC/Kevlar, (**d**) 7.5% nano-BC/Kevlar, and (**e**) 10% nano-BC/Kevlar.

The three-dimensional AFM surface topography images for the unmodified Kevlar and nano-BC/Kevlar membranes are illustrated in Figure 4. Figure 4a confirmed the relatively smooth morphology of the pure Kevlar membrane, which had been identified by the results of SEM. The surface morphology of the membranes changed significantly due to the presence of nano-BC, as characterized by the AFM results in Figure 4b–e. Comparatively, the 2.5% nano-BC/Kevlar membrane shows similar morphology to the pure Kevlar membrane without nano-BC decoration, which indicates that limited nano-BC (2.5%) could not significantly change the morphology of the membrane. In addition, the nano-BC could maintain a uniform dispersion in the membrane matrix without serious aggregation. Moreover, the surface roughness of 7.5% and 10% nano-BC/Kevlar membranes increased a lot. Comparatively, the surface morphology of the 5% nano-BC/Kevlar membranes appeared to be an affable structure and moderate mountain-like structure. Excessive (7.5% and 10%) nano-BC is prone to induce its agglomeration and shift the morphology of the Kevlar membrane significantly.

The surface roughness values, i.e., R_a_ (average roughness), R_rms_ (root mean square roughness), and R_m_ (maximum vertical difference between the highest and lowest points), are summarized in Table 2. The R_a_ for the pristine Kevlar, 2.5% nano-BC/Kevlar, and 5% nano-BC/Kevlar corresponds to 5.58, 6.58, and 6.97 nm, respectively, without a significant difference being found. For the membrane with 7.5% and 10% nano-BC, the membrane exhibited a rougher surface with an average roughness of 9.52 nm and 14.60 nm, respectively. The roughness value is consistent with the SEM and AFM images of the corresponding membranes.

#### 3.2.4. Water Contact Angle and MWCO of the Membrane

**Figure 5:**
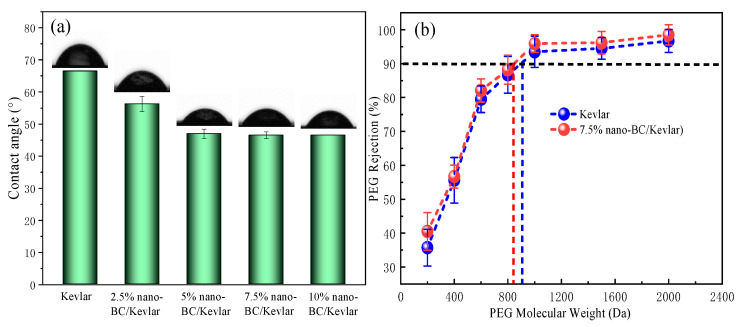
(**a**) The water contact angles of different membrane samples; (**b**) The MWCO of Kevlar and 7.5% nano-BC/Kevlar membranes.

The water contact angle (CA) values of the MMMs are shown in Figure 5a. The CA values of 2.5% nano-BC/Kevlar and 5% nano-BC/Kevlar membranes are 56.25° and 47.00°, respectively, which is obviously lower than that of the original membrane (66.50°). The CA of NF membranes declined with the increase in nano-BC doping content, which indicated the enhanced hydrophilicity of the membranes. As identified previously by XPS and FTIR, this result can be attributed to the instinct hydrophilic functional groups (i.e., -OH and -COOH) of nano-BC. Additionally, the nano-BC could also shift the CA by changing the surface roughness. Normally, a rougher surface possesses higher CA values due to the presence of trapping air between the solid surface and the liquid droplet. With a further increase in nano-BC to the membrane matrix, the MMMs maintained a stable CA result. This could be explained by the compensation effect of more functional groups introduced by nano-BC and the rougher surface. In addition, the enhanced interaction of Kevlar fiber and nano-BC with a higher nano-BC content resulting in a relatively denser structure could be another factor influencing the hydrophilicity [41]. From Figure 5b, the Kevlar membrane exhibited a MWCO around 900 Da. In the meantime, a similar MWCO for 7.5% nano-BC/Kevlar membranes was observed. This confirms the coverage of BC by the Kevlar fiber.

#### 3.2.5. Separation Performance

**Figure 6:**
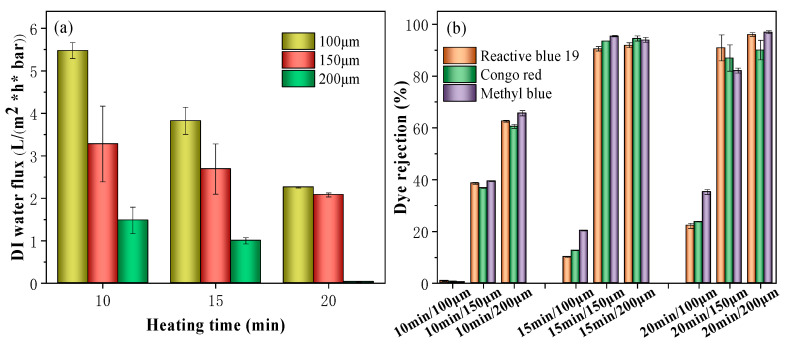
(**a**) Water flux and (**b**) the dye rejection of the Kevlar membrane with different thicknesses and heating times.

As shown in Figure 6, the results of water flux versus heating time of Kevlar membranes with different thicknesses were investigated. The results indicated that the Kevlar membrane with a thickness of 100 μm and 10 min heating time has the highest pure water flux of 5.47 L m^−2^ h^−1^ bar^−1^. However, an ultra-low dye rejection was obtained, with rejections of 0.88%, 0.59% and 0.42% for Reactive blue 19, Congo red, and methyl blue, respectively. When the thickness was adjusted to 150 μm, the dye rejection increased and the water flux decreased (Figure 6). When the thickness reached 200 μm, the rejection greatly improved by sacrificing the flux. The sharp decrease in pure water flux is mainly due to the increase in mass transfer resistance when the Kevlar thickness increased. For membranes with a heating time of 15 and 20 min, a similar trend was observed. For membranes with a thickness of 100 μm, a limited rejection under 80% for all three dyes was achieved, while membranes with a thickness of 200 μm obtained an ultra-low water permeance. Therefore, the optimized fabrication parameters were fixed at a heating time of 15 min with a casting thickness of 150 μm.

**Figure 7:**
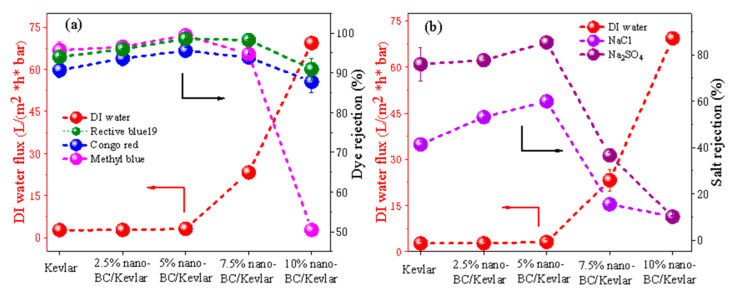
Effects of the nano-BC content on the flux and rejection ((**a**) dyes and (**b**) salts) of the nano-BC/Kevlar NF membranes.

The effects of nano-BC content on the Kevlar membrane NF performance are shown in Figure 7. Notably, compared to the pure Kevlar, 2.5% nano-BC MMMs have limited influence on the dye retention and pure water flux. However, the nano-BC/Kevlar membranes with 5% nano-BC showed an obvious enhancement in dye retention compared with neat Kevlar membranes (95.55%, 98.54%, and 99.41% for Congo red, Reactive blue 19, and methyl blue, respectively). As mentioned above, the interaction between nano-BC and Kevlar fiber could improve the compactness of the membrane [41]. Thus, a slight increase in the dye rejection was obtained. For MMMs with 7.5% nano-BC, the dye rejection of the composite Kevlar membrane is slightly reduced due to the formation of un-selective aggregates, especially for methyl blue with a rejection of 94.69%. Nevertheless, the flux was notably strengthened to 23.21 L m^−2^ h^−1^ bar^−1^. For membranes with 10% nano-BC, the dye rejection of the composite Kevlar membrane continued to reduce until a methyl blue rejection around 50.00% was achieved. Here, the Kevlar fiber served as the blocker to maintain the dye rejection and the supporter to sustain the membrane stability, while the nano-BC provided the extra channels for enhanced permeance.

Figure 7b displays the rejections of these membranes against NaCl and Na_2_SO_4_ solutions. It is obvious that the MMMs with different nano-BC contents found it hard to retain all the salts. The rejection follows an order of R (Na_2_SO_4_) > R (NaCl), which is consistent with the literature data [12,42]. As expected, the negatively charged nano-BC provided the membrane with a higher electro-repulsion effect towards SO_4_^2−^. Thus, the rejection for Na_2_SO_4_ was better than the NaCl. The revolution of the salt rejections was analogous with those of dye rejections. Typically, the membrane used for dye wastewater treatment should maintain a high water flux, enhanced dye rejection and low salt rejection. To gain a better performance, the membrane with 7.5% nano-BC could provide the membrane with excellent preparation performance towards dyes/salt solutions.

#### 3.2.6. Long-Term Stability Test and Its Comparison to Other Reported Membranes

**Figure 8:**
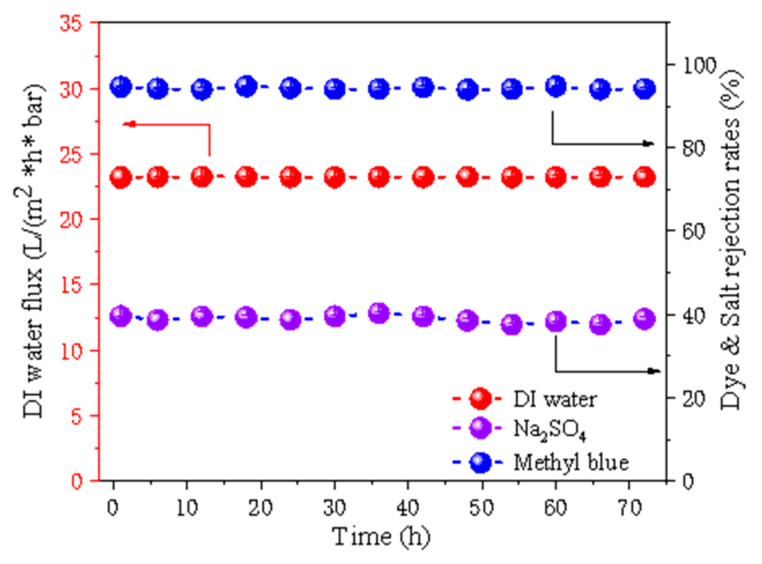
The long-time stability of 7.5% nano-BC/Kevlar membrane.

To effectively recover the stability of the membrane, the filtration tests of MMMs with a nano-BC content of 7.5% were performed for 72 h to evaluate the long-term operation stability. As shown in Figure 8, the pure water flux of the 7.5% nano-BC/Kevlar membrane maintained stability after 72 h operation. In the meantime, the membrane held a high methyl blue rejection (>91%), while the NaCl rejection was persistent at around 36.0% during the testing period. The results indicated that the nano-BC/Kevlar MMMs possess an excellent structure and performance stability, which provides the membrane with great application potential for textile wastewater treatment.

Table 3 lists the separation properties of the reported synthesized membranes and the nano-BC/Kevlar MMMs fabricated in this work concerning the pure water flux and dye rejection. In this study, the nano-BC/Kevlar MMMs with 7.5% nano-BC insertion are superior to most of the reported membranes concerning the relatively higher water permeability of 23.2 L m^−2^ h^−1^ bar^−1^. However, the dye rejection is comparatively lower than the reported membranes. Despite this, the MMMs still reached an excellent dye rejection of 93.9%, 98.2% and 94.7% for Congo red, Reactive blue 19 and methyl blue, respectively. Consequently, the obtained MMMs with excellent water flux and high dye retention are suitable for the application of dye removal.

## 4. Conclusions

Kevlar and nano-BC were successfully introduced to fabricate the composite NF membrane by a thermal-assisted phase inversion method. By omitting the traditional interfacial polymerization, the fabrication process was greatly simplified. The Kevlar in the membrane matrix ensured the MMMs’ high dye rejection while the nano-BC facilitated the permeability. Favorable physical/chemical structures and properties of the membrane were additionally identified by a series of characterizations. At optimal fabrication conditions, the membranes displayed high dye (e.g., Congo red, Reactive blue 19 and methyl blue) retentions and excellent water permeability, as well as low salt permeation. Due to the extra-stability of Kevlar fiber, the nano-BC/Kevlar MMMs preserved their rejections and water flux. The environmentally friendly and easily accessible characteristics of BC supply a good material for the industrialization of membrane synthesis. This work has laid a foundation for the preparation of heating-assisted NF membranes, and provided some guidance for the application of Kevlar fibers and BC in membrane preparation.

## Figures and Tables

**Table 1:** Elemental composition, atomic ratio, and polar index of nano-BC by elemental analysis (EA) and XPS.

Method	Ash (%)	C (wt%)	N (wt%)	H (wt%)	O (wt%)	S (wt%)	Si (wt%)	Ca (wt%)	O/C	H/C	(O + N)/C
EA	7.01	69.61	2.70	3.04	16.65	0.99	ND	ND	0.18	0.52	0.21
XPS	-	67.78	2.36	-	26.95	-	2.16	0.76	0.30	ND	0.33

**Table 2:** AFM parameters of the pristine and modified membranes.

Membrane	R_a_ (nm)	R_rms_ (nm)	R_m_ (nm)
Kevlar	5.58	7.41	58.30
2.5% nano-BC/Kevlar	6.58	8.43	66.30
5% nano-BC/Kevlar	6.97	8.62	83.70
7.5% nano-BC/Kevlar	9.52	11.80	37.80
10% nano-BC/Kevlar	14.60	17.80	98.30

**Table 3:** Performance of different membranes reported by the literature.

Membrane	Dye	Dye Rejection (%)	Flux (L/(m^2^·h·bar))	Reference
(PEI- GO)/PAA/PVA/GA	Methyl blue	99.3	0.8	[43]
(PAA/PEI)5	Methyl blue	>99.0	1.7	[44]
(PDDA/PAA)/PAN	Methyl blue	99.3	3.4	[45]
PES-TA	Methyl green	98.0	2.0	[46]
(CMCNa/PEI)/PP	Congo red	99.4	5.7	[47]
ZIF-8/PES	Congo red	92.5	5.0	[48]
BHAC/PIP	Methyl blue	98.9	8.5	[49]
(TA/Fe3+)/P84	Methyl blue	95.0	9.8	[50]
(TA/TMC)/PES	Congo red	99.8	16.8	[51]
M−7	Congo red	99.6	40.6	[52]
	Reactive black 5	99.5		
	Reactive orange 16	96.2		
Nano-BC/Kevlar	Congo red	93.9	23.2	This work
	Reactive blue 19	98.2		
	Methyl blue	94.7		

## Data Availability

Not applicable.
